# ECOA-6. Genomic and transcriptomic analyses reveal diverse mechanisms responsible for deregulation of epigenetic enzyme/modifier expression in glioblastoma

**DOI:** 10.1093/noajnl/vdab070.006

**Published:** 2021-07-05

**Authors:** Marta Maleszewska, Bartosz Wojtas, Bartlomiej Gielniewski, Shamba Mondal, Jakub Mieczkowski, Michal Dabrowski, Janusz Siedlecki, Mateusz Bujko, Pawel Naumann, Wieslawa Grajkowska, Katarzyna Kotulska, Bozena Kaminska

**Affiliations:** 1 Nencki Institute of Experimental Biology, Warsaw, Poland; 2 The Maria Sklodowska-Curie National Research Institute of Oncology, Warsaw, Poland; 3 The Children’s Memorial Health Institute, Warsaw, Poland

## Abstract

Malignant gliomas represent over 70% of primary brain tumors and the most deadly is glioblastoma (GBM, WHO grade IV), due to frequent dysfunctions of tumor suppressors or/and oncogenes. Recent whole genome studies of gliomas demonstrated that besides genetic alterations, epigenetic dysfunctions contribute to tumor development and progression. Alterations in genes encoding epigenetic enzyme/protein or aberrations in epigenetic modification pattern have been found in gliomas of lower grade, yet no epigenetic driver was identified in GBM. We sought to identify different mechanisms driving aberrant expression of epigenetic genes in GBM.

We analyzed gene expression and coding/non-coding regions of 96 major epigenetic enzymes and chromatin modifiers in 28 GBMs, 23 benign gliomas (juvenile pilocytic astrocytomas, JPAs, WHO grade I) and 7 normal brain samples. We found a profound and global down-regulation of expression of most tested epigenetic enzymes and modifiers in GBMs when compared to normal brains and JPAs. For some genes changes in mRNA level correlated with newly identified single nucleotide variants within non-coding regulatory regions. To find a common denominator responsible for the coordinated down-regulation of expression of epigenetic enzymes/modifiers, we employed PWMEnrich tool for DNA motif scanning and enrichment analysis. Among others, we discovered the presence of high affinity motifs for the E2F1/E2F4 transcription factors, within the promoters of the epigenetic enzyme/modifier encoding genes. Knockdown of the E2F1/E2F4 expression affected the expression of a set of epigenetic enzymes/modifiers. Altogether, our results reveal a novel epigenetic-related pathway by which E2F1/E2F4 factors contribute to glioma pathogenesis and indicate novel targets for glioma therapy.

Supported by a National Science Centre grant 2013/09/B/NZ3/01402 (MM).

